# The Insight into Insulin-Like Growth Factors and Insulin-Like Growth-Factor-Binding Proteins and Metabolic Profile in Pediatric Obesity

**DOI:** 10.3390/nu13072432

**Published:** 2021-07-15

**Authors:** Wojciech Czogała, Wojciech Strojny, Przemysław Tomasik, Mirosław Bik Multanowski, Małgorzata Wójcik, Klaudia Miklusiak, Emil Krzysztofik, Albert Wróbel, Karol Miklusiak, Szymon Skoczeń

**Affiliations:** 1 Department of Pediatric Oncology and Hematology, University Children’s Hospital of Krakow, 30-663 Krakow, Poland; czogala@tlen.pl (W.C.); wojciech.strojny@mp.pl (W.S.); 2 Department of Clinical Biochemistry, Faculty of Medicine, Jagiellonian University Medical College, 30-663 Krakow, Poland; p.tomasik@uj.edu.pl; 3 Department of Medical Genetics, Faculty of Medicine, Jagiellonian University Medical College, 30-663 Krakow, Poland; miroslaw.bik-multanowski@uj.edu.pl; 4 Department of Pediatric and Adolescent Endocrinology, Faculty of Medicine, Jagiellonian University Medical College, 30-663 Krakow, Poland; malgorzata.wojcik@uj.edu.pl; 5 Student Scientific Group of Pediatric Oncology and Hematology, Jagiellonian University Medical College, 30-663 Krakow, Poland; klaudia.mklk@gmail.com (K.M.); emilkrzysztofik.ek@gmail.com (E.K.); albus54@poczta.fm (A.W.); karolmiklusiak@gmail.com (K.M.); 6 Department of Pediatric Oncology and Hematology, Faculty of Medicine, Jagiellonian University Medical College, 30-663 Krakow, Poland

**Keywords:** IGF, IGFBP, obesity, children, microarray

## Abstract

Insulin-like growth factors (IGFs) and insulin-like growth-factor-binding proteins (IGFBPs) regulate cell proliferation and differentiation and may be of importance in obesity development. The aim of the study was to analyze the expression of chosen IGF-axis genes and the concentration of their protein products in 28 obese children (OB) and 34 healthy control (HC), and their correlation with essential parameters associated with childhood obesity. The gene expression of IGFBP7 was higher, and the expression of IGF2 and IGFBP1 genes was lower in the OB. The expression of IGFBP6 tended to be lower in OB. IGFBP4 concentration was significantly higher, and IGFBP3 tended to be higher in the OB compared to the HC, while IGFBP1, IGFBP2, and IGFBP6 were significantly lower, and IGFBP7 tended to be lower in OB. We found numerous correlations between IGFs and IGFBP concentration and obesity metabolic parameters. IGFBP6 correlated positively with apelin, cholecystokinin, glucagone-like peptide-1, and leptin receptor. These peptides were also significantly lower in obese children in our study. The biological role of decreased levels of IGFBP6 in obese children needs further investigation.

## 1. Introduction

Obesity is a complex condition with a serious impact on overall health, both physical and psychological. It is defined by the World Health Organization (WHO) as “abnormal or excessive fat accumulation that may impair health” [1]. It is commonly known that obesity leads to many diseases, including cancer [2,3,4], and has a prominent role in the pathogenesis of type 2 diabetes in adolescents and adults [5]. Obesity remains a constant threat to overall health by also causing several other medical conditions. At any age, obesity can affect the cardiovascular, respiratory, skeletal, and endocrine systems [6,7]. Importantly, it not only affects the physical sphere, but also negatively affects the psyche and self-esteem [8]. This common condition is now regarded as a very widespread and growing problem in pediatrics and has been referred to as “an epidemic” [9,10].

Until now, the role of the insulin-like growth factors’ axis and the individual markers and hormones associated with obesity in children are poorly studied [4,11,12]. Insulin-like growth factor (IGF) provides cells with information on the well-being of the body, it regulates proliferation, differentiation, and synthesis. This kind of signaling plays high importance in the growth of the organism, but also in neoplastic processes as well as in development of obesity. Particularly in neoplasm with very poor prognosis, the IGF-axis involvement should be explored [13]. The IGF system consists of modulatory proteins IGF1 and IGF2, which interact at the cellular level with the insulin-like growth factor receptor (IGFR). This pathway also includes regulatory proteins, known as insulin-like growth-factor-binding proteins (IGFBPs) that regulate IGF signaling. Extensive experiments in animal models demonstrate that adipose tissue expansion induces a complex and broad immune response, involving both the innate and adaptive arms of the immune system, playing critical roles in the regulation of glucose metabolism and inflammation [14]. The important role of proinflammatory cytokine secretion from adipose tissue has been consistently associated with the risk of adverse outcomes in obesity-linked complications, promoting a persistent, low-grade, inflammatory response. Accordingly, adipokines are considered as regulators of whole-body homeostasis [15]. However, very little is known about the above-mentioned mechanisms in children in the context of obesity.

Because the IGF protein family plays a key role in metabolic processes, we decided to examine the differences in the concentration of numerous components of the IGF axis (IGF1, 2, IGFBP 1, 2, 3, 4, 6, 7) in obese children in comparison to healthy control and to analyze the correlations of these components with parameters, such as blood pressure, concentration of insulin, adipokines (adiponectin, apelin, resistin, visfatin, leptin, leptin receptor), and peptides regulating gastrointestinal tract (cholecystokinin, ghrelin, GLP-1, FGF21). The peptides concentration was assessed both fasting and after an oral glucose tolerance test. Additionally, taking into account the importance of epigenetic factors, the expression of genes regulating studied peptides of the IGF axis were analyzed.

## 2. Materials and Methods

### 2.1. Study Group

Two study groups were recruited. The obesity group (OB) included 28 children, 12 boys and 16 girls, 4–17.8 (average 13.7) years old. All were patients of the Pediatric and Adolescents Endocrinology Department, Jagiellonian University, Medical College in Krakow. Obesity was defined as a BMI at or above the 95th percentile for children of the same age and sex. BMI was calculated by dividing a children’s weight in kilograms by the square of height in meters. The inclusion criteria were obesity (BMI Z-score > 2.0) developed before the age of puberty and negative medical history or any signs or symptoms of acute or chronic diseases, no drugs and dietary supplements, and normal diet. The exclusion criteria were obesity secondary to medical conditions (single gene mutations, endocrinopathies), chronic systemic diseases, or drugs (e.g., glucocorticosteroids).

The control group consisted of 34 healthy peers: 13 boys and 21 girls, aged 4.3–16.9 (average 11.8) years. The children were recruited from the families of the patients and children of medical staff, analogous to the study group in terms of age and sex, all having a negative medical history and without any signs or symptoms of acute or chronic diseases, including obesity.

### 2.2. Anthropometric Evaluation

Height and body weight measurements were performed by an anthropometrist. Weight was measured to the nearest 0.1 kg, and height was measured to the nearest 0.1 cm using a stadiometer and a balanced scale. The body mass index (BMI) and BMI percentile/SDS were calculated using online WHO BMI calculators based on CDC growth charts for children and teens ages 2 through 19 years. The results were compared to regional reference values (WC) and values published by WHO (BMI percentile/SDS).

### 2.3. Protocol of the Study

Blood was taken morning in the fasting state. Blood concentrations of glucose, insulin, adiponectin, apelin, cholecystokinin, fibroblast growth factor 21, glucagon-like peptide-1, leptin, leptin receptor, resistin, and visfatin were measured at fasting as well as at 60 and 120 min of the standard oral glucose tolerance test (OGTT) performed using 1.75 g of anhydrous glucose per kilogram of body weight (maximum of 75 g). Collection was performed once after selection to the study. The samples were collected in tubes containing aprotinin. The material was immediately delivered to the laboratory at +4 °C and centrifuged for 15 min at a relative centrifugal force of 1590× *g*. Plasma samples for insulin, total IGF1 and IGF2, IGFBP1, -2, -3, -4, -6, and -7 analysis were stored at −80 °C until the time of the assay.

### 2.4. Biochemical Tests

Fasting insulin concentrations were measured with immunoradiometric kits (BioSource Company Europe S.A). The concentrations of total IGF and BP were measured using ELISA kits as follows:

- IGF1—Labor Diagnostika Nord GmbH & Co.KG, Germany,
- IGF2—Mediagnost, Reultingen, Germany,
- IGFBP1, IGFBP2, IGFBP3, IGFBP6—Mediagnost, Reultingen, Germany,
- IGFBP4, IGFBP7—FineTest, Wuhan, China.

### 2.5. Microarray Analysis

We assessed the whole genome expression in peripheral blood leukocytes using GeneChip Human Gene 1.0 ST Array (Affymetrix, Santa Clara, CA, USA). Total RNA extraction was performed using RiboPure Blood Kit (Ambion, Life Technologies, Carlsbad, CA, USA). The whole transcript microarray experiment was performed according to the manufacturer’s protocol (GeneChip Whole Transcript sense Target Labeling Assay Manual, Version 4).

### 2.6. Statistical Analysis

Continuous clinical and biochemical variables were presented as mean or median as appropriate. The Shapiro–Wilk test was used to assess the normality of continuous variables. To examine the differences between two independent groups, the Student’s *t*-test (for normally distributed variables) or Mann–Whitney test (for non-normally distributed variables) were used. Two-sided *p*-values < 0.05 were considered statistically significant. To assess the correlations between 2 continuous variables, Spearman’s rank correlation coefficient was calculated. Two-sided *p* values < 0.05 were considered statistically significant. Gene expression data were robust multi-array average (RMA)-normalized and presented as mean and standard deviation. ANOVA was used to examine the differences in gene expression between 2 independent groups. Benjamini–Hochberg (B–H)-corrected *p* values < 0.05 were considered statistically significant. Statistical analysis was performed using Statistica 13.3 (Statsoft Inc., Tulsa, OK, USA).

The Permanent Ethical Committee for Clinical Studies of the Medical College of the Jagiellonian University approved the study protocol (consent number KBET/249/B/2013 26 October 2013). All parents, adolescent patients, and adult patients signed a written informed consent before blood sample collection. The study conforms with The Code of Ethics of the World Medical Association (Declaration of Helsinki), printed in the British Medical Journal (18 July 1964).

## 3. Results

The characteristics of study group was presented in Table 1, and the values of metabolic parameters were shown in Table 2.

### 3.1. Concentration of IGF Proteins

The differences in the mean concentrations of the IGF-axis proteins were presented in Table 3. Mean concentrations of IGFBP3 and IGFBP4 in the obesity group (OB) were higher than in the control group (HC). The differences were significant (*p* < 0.05) for IGFBP4; there was a trend for IGFBP3. However, the median value of IGF2, IGFBP1, IGFBP2, IGFBP6, and IGFBP7 were lower in the OB group than in the HC group, and the differences were significant for all the parameters (*p* < 0.001) except for IGF2 and IGFBP7, where the trend was observed.

### 3.2. IGF Proteins Concentration and Other Metabolic Parameters

**Figure 1:**
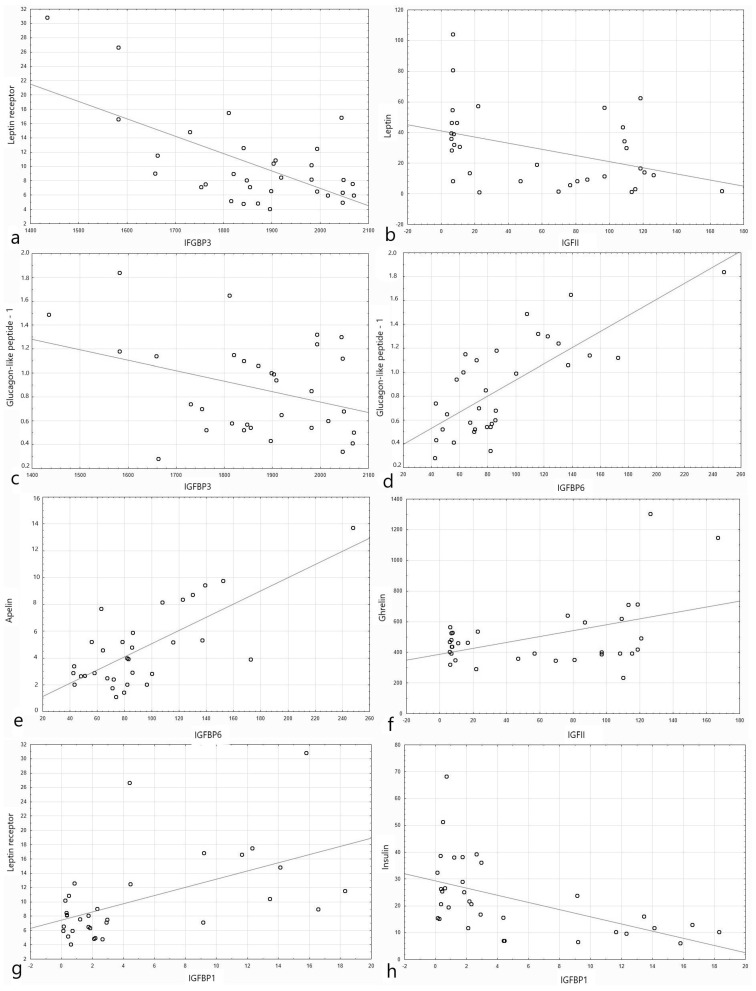
Plots presenting the distribution of data of the studied parameters depending on the level of different IGF family proteins’ level (shown as log2 of the absolute value of blood level for clarity of the plot). (**a**) Leptin receptor (µg/L) depending on the level of IGFBP3 (ng/mL). (**b**) Leptin (ng/dL) depending on the level of IGF2 (ng/mL). (**c**) Glucagon-like peptide-1 (nmol/dL) depending on the level of IGFBP3 (ng/mL). (**d**) Glucagon-like peptide-1 (nmol/dL) depending on the level of IGFBP6 (ng/mL). (**e**) Apelin (pg/mL) depending on the level of IGFBP6 (ng/mL). (**f**) Ghrelin (pg/mL) depending on the level of IGF1 (ng/mL). (**g**) Leptin receptor (µg/L) depending on the level of IGFBP1 (ng/mL). (**h**) Insulin (µIU/mL) depending on the level of IGFBP1 (ng/mL).

The correlation results of IGF1 and IGF2 proteins’ concentrations with selected metabolic parameters are presented in Table 4, while the correlation results of IGFBPs’ concentrations are shown in Table 5. Plots presenting the distribution of chosen data of the studied parameters depending on the level of different IGF family proteins’ level are presented in Figure 1. The study revealed a statistically significant positive correlation between BMI and IGFBP3 and negative with IGFBP6 and IGFBP1. Blood pressure was positively correlated with IGFBP3 and negatively with IGFBP-1 and IGFBP2. The fasting insulin blood level and OGTT was positively correlated with IGFBP4 and negatively with IGFBP1, IGFBP2, and IGFBP3. There was also a negative correlation between the fasting insulin level and IGFBP6. There were no significant correlations between adiponectin and the proteins of the IGF axis. Apelin was positively correlated with IGF2, IGFBP6, and also, IGFBP-3 (for the last protein, the results were borderline). Cholecystokinin was positively correlated with IGF2, IGFBP6, and IGFBP7 (for the last protein, the results were borderline) and negatively with IGFBP3.

Fibroblast growth factor 21 was positively correlated with IGFBP6. Ghrelin was positively correlated with IGF2, IGFBP1, and IGFBP2 and negatively with IGFBP4. Leptin was negatively correlated with IGF2, IGFBP1, and IGFBP2. The leptin receptor was positively correlated with IGFBP1, IGFBP2, and IGFBP6 and negatively with IGFBP3. For IGF2, the correlation was positive but not significant. The level of resistin in the 60th and 120th minute correlated positively with IGFBP2 and negatively with IGFBP3. Visfatin revealed a positive correlation with IGFBP4, IGFBP6, and IGFBP7.

### 3.3. Expression of IGF Proteins’ Genes

**Figure 2:**
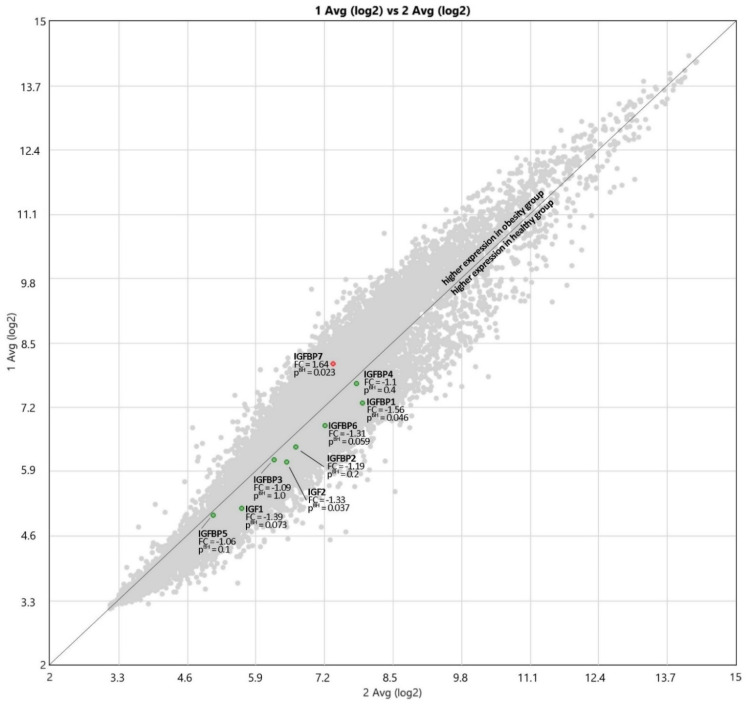
The expressions of IGF and IGFBP proteins’ genes; 1 Avg (log2)—OB, 2 Avg (log2)—HC, FC—Fold Change.

**Figure 3:**
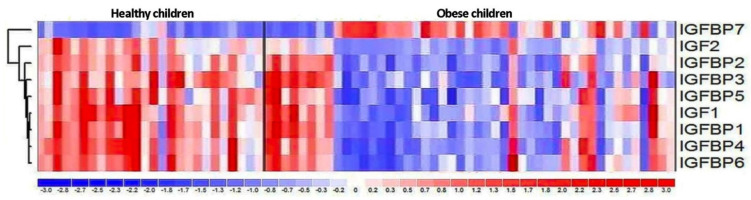
The hierarchical clustering showing differences in the expression patterns of IGF-axis genes between healthy control and obese groups.

The expressions of IGF proteins’ genes are presented in Figure 2, while the expression values are presented in Table 6. The hierarchical clustering showing differences in the expression patterns of IGF-axis genes between healthy control and obese groups is presented in the Figure 3. A comparison of the obesity and control groups revealed differences in the expression of IGF2, IGFBP1, and IGFBP7 genes. The expression of IGFBP7 was higher (*p* = 0.023), and the expression of IGF2 (*p* = 0.037) and IGFBP1 (*p* = 0.046) genes were lower in the OB group. IGFBP6 gene expression tended to be lower (*p* = 0.059) in the OB group.

## 4. Discussion

In our study, we showed that the concentration of IGF-axis proteins differs in the healthy and obese pediatric population, as well as numerous, statistically significant correlations between the concentration of the studied proteins and the concentration of adipokines, gastrointestinal tract hormones, insulin or blood pressure. Additionally, we showed statistically significant differences in gene expression of IGF proteins’ family between obese and healthy children.

### 4.1. Concentration of IGF-Axis Proteins

In the study, we noted the significant differences in IGF-axis proteins’ concentration between the group of obese and healthy children, which may play a role in the pathogenesis of obesity. IGFBP4 showed significantly higher values, and there was a trend toward a higher concentration of IGFBP3 in the OB compared to the HC, while IGFBP1, IGFBP2, and IGFBP6 achieved significantly lower values, and IGFBP7 tended to be lower in OB. No significant difference in the median concentration of IGF1 protein in the serum of obese children and in healthy children was found. The currently published results are inconsistent showing higher [4,16], lower [12,17,18,19], and comparable [20,21,22] values of IGF1 in obese individuals in comparison to control of normal weight, both in pediatric and adult population. The main but not only factor influencing the production of IGF1 is a growth hormone (GH). In obese people, its reduced level and the lower response to factors increasing its secretion (e.g., physical activity) have been repeatedly demonstrated [23,24,25]. However, no sufficient explanation of the changes in total serum IGF1 concentration of obese patients in response to decreased GH level can be drawn, especially that despite the decreased GH levels, obese patients grew properly [26].

In the group of obese children, the concentration of IGF2 was lower almost significantly (*p* = 0.06) than in the control group. This protein plays an important role in the fetus growth regulation. Increased secretion is believed to be responsible for overgrowth of the fetus and increased amount of adipose tissue [27]. Alfares et al. suggested that IGF2 may stimulate subcutaneous preadipocyte differentiation and inhibit visceral preadipocyte differentiation [28]. However, its impact on obesity in older children is not sufficiently understood, and there is a need for further research in this area.

IGF binding proteins (IGFBPs) are a family of structurally similar proteins that are responsible for transport, extension of half-life, regulation of clearance, and direct modulation of IGF activity [29]. IGFBP1 concentration in our study group of obese children was significantly lower than in the control group. Similar results can be found in other publications [30,31,32,33]. It was initially suggested that a reduced concentration of IGFBP1 might increase the level of free IGF1 to compensate for the decreased concentration of GH in obese subjects. However, the results of research in this area are ambiguous [24]. A similar result was obtained for IGFBP2, the concentration of which in our study was significantly lower among obese patients. Jung Min Ko et al. also received significantly reduced levels of this protein in obese children [34].

IGFBP3 is the most abundant protein in the serum and is responsible for the transport of 90–95% of IGF1 and IGF2 [29]. This protein, apart from transport functions, is in charge for the amount of IGF available for receptors. Structural changes in IGFBP3 affect the amount of free IGF. IGFBP3 concentration, especially in children, is related to GH concentration [35]. Our results showed the trend toward higher concentration of IGFBP3 in obese children compared to the control group, what was consistent with the data from literature [22,36], and the results with not significant differences were also described [4]. However, Ounis O.B. et al. showed that IGFBP3 concentrations were significantly reduced following a diet or exercise associated with weight loss [37], and Juul A. et al. found that IGFBP3 levels increase with age and peak at puberty [38].

In our study, IGFBP4 had a significantly higher concentration in obese patients than in non-obese children. The liver is the main place of production of this protein, but its presence has also been demonstrated in other tissues [39,40]. IGFBP4 expression appears to play, similar to IGF2, an important role in the early growth period. A study in mice shows that in the absence of IGFBP4 production, the mice were born smaller than the controls, and this difference was maintained later in life [41]. It has also been shown that local excess IGFBP4 has a negative effect on the growth of smooth muscle [42]. However, the systemic administration of IGFBP4 had a stimulating effect on the process of bone formation [43]. It is presumed that in the absence of IGFBP4, IGF factors are more likely to be degraded. On the other hand, the significantly increased concentration of IGFBP4 exceeds the capabilities of proteolytic proteins, which are responsible for the amount of active IGF [41].

IGFBP6 has a much greater affinity for IGF2 than for IGF1 [44]. Therefore, it mainly inhibits IGF2 activity, but it may also have IGF-independent actions [45]. The IGFBP7 is believed to influence cell growth processes in the body; however, its affinity for IGF is significantly lower compared to IGFBP1, -2, -3, -4, -5, and -6 [46]. In our study, the concentration of IGFBP6 turned out to be significantly lower in obese children compared to healthy ones, and there was trend toward lower IGFBP7 level. However, the concentrations of these proteins have not yet been described in the context of childhood obesity.

Additionally, the IGFBPs undergo numerous post-translational modifications that may change their properties, and they are sensitive to the action of various types of proteases [47]. It has also been proven that they show numerous activities independent of IGF [48]. Differences in IGF receptor expression may also be important in assessing the influence of the IGF axis on growth processes. Ricco et al. showed higher IGF-1R mRNA expression among obese children [49]. The issue of the mutual actions of GH, IGF, and various IGFBPs on each other and the body’s cells seems to be quite complex and not fully understood at the moment. Many publications are not consistent, and some elements of the GH-IGF axis are not sufficiently studied. Existing differences may result from the method of measurement and the influence of other factors involved in the regulation of metabolism. Therefore, there is a need for further studies that will focus not only on the GH-IGF-axis proteins, which are poorly understood, but also on a more thorough assessment of possible dependencies and causality of existing trends.

### 4.2. IGF Proteins Concentration and Other Metabolic Parameters

In our study, the correlations of selected parameters related to obesity and other IGF-axis proteins were also assessed. All the correlations we describe is statistically significant. However, they differ in terms of strength (as presented in Table 4 and Table 5). Hence, further research is needed to better understand and define the presented dependences, as well as to determine the relevance of our findings. BMI correlated negatively with IGFBP6. The research shows that this protein is a comparatively specific inhibitor of IGF2 actions [50]. We speculate that the low level of IGFBP6 in obese patients cannot decrease the level IGF2 and that may be the factor that contributes to obesity. Moreover, IGFBP6 correlated positively with apelin, cholecystokinin, glucagone-like peptide-1, and leptin receptor. These peptides were also significantly lower in obese children in our study (Table 2). The data in literature about the role of IGFBP6 in obesity in children are scarcely available. Therefore, the biological role of a decreased level of IGFBP6 in obese children needs further investigations.

Systolic blood pressure correlated negatively with the concentration of IGFBP1 and IGFBP2. However, the values of Spearman’s correlation coefficient were moderate. Children with obesity have several times higher risk of hypertension in comparison to children with normal weight. Moreover, the risk increases with BMI value [51]. It is possible that different IGF2 gene variants affect blood pressure regulation in obese children [52]. Studies in the adult population showed that different IGFBP1 gene variants may have an effect on blood pressure, and the concentration of IGFBP1 in the serum of people with hypertension was lower compared to the healthy individuals [53]. This suggests a possible relevance of these proteins as possible blood pressure-related biomarkers, also in children.

Insulin correlated positively with IGFBP3 in OGTT and negatively with IGFBP1 and IGFBP2. However, other studies also indicate the positive correlation between IGFBP3 and insulin level [54]. It seems likely that IGFBP1 and IGFBP2 have a beneficial effect on the risk of developing diabetes [55,56]. In other studies, negative correlations between insulin and IGFBP1 as well as insulin and IGFBP2 were also shown [22,33,57]. Therefore, appropriate concentrations of the above proteins of the IGF axis may play an important role in maintaining the normal level of glucose in the body. On the other hand, insulin can affect the number of individual components of the IGF axis.

Both IGFBP1 and IGFBP2, and also IGF2 in OGTT, negatively correlated with leptin. However, for the leptin receptor, we showed a negative correlation with IGFBP3 and a positive correlation with IGFBP1, IGFBP2, and IGFBP6. Higher levels of leptin are characteristic of obese children. In addition, it is possible that leptin contributes to the achievement of normal growth in obese children, despite the lowered levels of growth hormone, by acting directly on growth cartilage cells and indirectly through the components of the IGF-axis proteins [58]. Ibarra-Reynoso et al. also noted a negative correlation between leptin and IGFBP1 [59]. The study in the obese pediatric population suggests that IGFBP2 may be a local preventive factor in adipose tissue accumulation. In the adipose tissue of obese people, compared to the healthy ones, the concentration of IGFBP2 mRNA is lower [60]. Perhaps the documented effect of leptin inducing IGFBP2 mRNA expression and IGFBP2 production is not fully effective due to leptin resistance found in obese subjects [61]. The concentration of a soluble leptin receptor also has a significant effect. Its increased level has been shown to bind leptin and inhibit its action. However, the reduced amount may reflect a low level of membrane leptin receptor expression, which may influence the state of leptin resistance [62]. Cinaz et al. showed a significantly lower concentration of soluble leptin receptor in obese children compared to healthy children [63]. In our study, the correlations of IGFBP1 and IGFBP2 and the leptin receptor were opposite to the correlation of these proteins with leptin concentration; hence, leptin appears to play an important role in IGF-axis regulation.

In the case of ghrelin, we showed a positive correlation with IGF2, IGFBP1, and IGFBP2, both fasting and after glucose administration, IGFBP1 only after OGTT, and a negative correlation with IGFBP4 also after OGTT. It is worth mentioning that despite the statistical significance, these correlations were not strong, but moderate. Önnerfält et al. showed lower levels of ghrelin among obese children and noticed a negative correlation between the fasting ghrelin level and the body mass index, which is consistent with our results [64]. Ghrelin is a circulating orexigenic factor of which the level is reduced in obese humans [65]. It is suggested that the changes in the concentration of this peptide may be an adaptive response to the increase in body weight and the amount of adipose tissue [66]. Additionally, we noted that cholecystokinin and glucagon-like peptide-1 correlated positively with IGFBP6.

### 4.3. Expression of IGF Proteins’ Genes

Comparing the mRNA expression of the IGF-axis proteins, we obtained statistically significantly lower values among obese children of IGF2 and IGFBP1; IGFBP6 tended to be lower (*p* = 0.059) in the OB group. On the other hand, higher expression was noted in the case of IGFBP7 protein. At present, in the literature, there are no comprehensive data on the expression of the IGF-axis genes in humans. The standard source of diagnostic material is peripheral blood, which is readily available. Mononuclears show high expression of genes involved in lipid homeostasis and rapidly detect signals of its disturbance [67]. These genes’ expressions might be potential biomarkers of lipid metabolism abnormalities.

The study carried out in rats regarding IGF1 expression showed different results of the intensity of expression in obese individuals depending on the type of tissue [68]. Another study in obese adults showed a significantly lower value of IGF-1Eb mRNA isoform in muscle cells compared to the control group [69]. In mice, an association has also been demonstrated between decreased IGFBP1 mRNA expression and obesity [70].

A study in the adult population showed higher levels of IGFBP2 and IGFBP7 mRNA in obese subjects, and lower IGFBP4 mRNA expression. In our study, despite lower IGF-1 mRNA expression in obese children, we showed no significant difference in IGF-1 protein concentration. The decreased expression of IGF2, IGFBP1, and IGFBP6 corresponds to the decreased amount of these proteins in the group of obese children. In the case of decreased IGFBP7 concentration, we showed higher IGFBP7 mRNA expression. The above results indicate that the regulation of the transcription may be of greater importance for the level of IGF2, IGFBP1, and IGFBP6 than for other components of IGF axis. However, the mechanism of regulation of the concentration of proteins of the IGF family seems to be complex and, apart from epigenetic factors, also influenced by many additional factors.

There are few data in the literature that attempt to describe the relationship between obesity and IGF-axis proteins, e.g., in the papers by Saitoh et al. or Ballerini et al. [71,72]. However, these works are based only on the concentration of these proteins and not on their expression. Based on our preliminary observations, the role of the IGF axis in childhood obesity needs further investigations. It seems that a decreased level of IGFBP6 might play some role in obesity in the child population.

### 4.4. Limitations

The main limitation of the study was the small sample size, which could affect the validity of the results. In the future, it would be advisable to carry out similar experiments on larger cohorts to further confirm the obtained results. Certain outcomes, which did not reach the statistical significance cutoff value, are likely to be found significant when investigated with a population of greater size.

## 5. Conclusions

The study revealed relationship between IGF–axis proteins and gene expression and childhood obesity with its metabolic parameters. We suggest that the IGF axis may be involved in obesity development, but the exact mechanism cannot be distinctly defined based on the study. The relationship between GH, IGF, and IGFBPs, as well as their interactions with the body’s cells, is complex and remains not fully understood. We found numerous correlations between IGFBP6 concentration and obesity metabolic parameters. As the available data for the expression and concentration of IGF-family proteins are inconsistent, further research, concerning pediatric obesity, including larger populations, is necessary.

## Figures and Tables

**Table 1:** The characteristics of the study group. Values are presented as mean ± standard deviation.

Baseline Characteristic	Obesity Group (*n* = 28)	Control Group (*n* = 34)	*p* Value
Boys/girls *n* (%)	12/16 (42.9%/57.1%)	13/21 (38.2%/61.8%)	0.574
Age (years)	13.4 ± 3.8	12.0 ± 3.5	0.207
Height (cm)	168.89 ± 15.42	152.1 ± 20.2	0.090
Weight (kg)	86.5 ± 26.9	46.05 ± 17.85	<0.001
BMI (percentile)	99.6 ± 0.86	61.87 ± 28.6	0.002
BMI Z-score	2.30 ± 0.27	0.05 ± 0.89	<0.001
Blood pressure (average systolic)	125 ± 9.61	110 ± 9.82	<0.001
Blood pressure (average diastolic)	77 ± 6.76	66 ± 8.75	<0.001
HOMA	5.2 ± 3.8	2.3 ± 1.5	0.13

BMI-Body Mass Index; HOMA-Homeostatic Model Assessment.

**Table 2:** The metabolic parameters of the study groups. Values are presented as mean ± standard deviation.

Baseline Characteristic	Obesity Group (*n* = 28)	Control Group (*n* = 34)	*p* Value
Glucose T0 (mmol/L)	4.52 ± 0.74	4.65 ± 0.46	0.479
Glucose T60 (mmol/L)	6.64 ± 1.65	6.96 ± 1.8	0.529
Glucose T120 (mmol/L)	5.43 ± 1.59	5.67 ± 1.25	0.581
Insulin T0 (µIU/L)	25.82 ± 13.72	10.6 ± 4.6	<0.001
Insulin T60 (µIU/L)	162.16 ± 94.44	63.26 ± 39.77	<0.001
Insulin T120 (µIU/L)	113.42 ± 77.85	45.41 ± 24.91	<0.001
Adiponectin T0 (µg/mL)	2.79 ± 1.54	4.04 ± 2.19	0.037
Adiponectin T60 (µg/mL)	2.76 ± 1.78	4.29 ± 2.21	0.026
Adiponectin T120 (µg/mL)	2.62 ± 1.65	4.05 ± 2.11	0.011
Apelin T0 (pg/mL)	1.76 ± 0.79	4.32 ± 1.74	<0.001
Apelin T60 (pg/mL)	1.63 ± 0.83	4.15 ± 1.95	<0.001
Apelin T120 (pg/mL)	1.77 ± 1.06	4.04 ± 1.92	<0.001
Cholecystokinin T0 (nmol/L)	1.5 ± 1.06	3.9 ± 1.5	<0.001
Cholecystokinin T60 (nmol/L)	1.42 ± 0.87	3.46 ± 1.25	<0.001
Cholecystokinin T120 (nmol/L)	1.44 ± 0.92	3.87 ± 1.08	<0.001
Fibroblast growth factor 21 T0 (ng/mL)	169.65 ± 92.06	87.89 ± 48.17	<0.001
Fibroblast growth factor 21T60 (ng/mL)	137.08 ± 70.6	77.18 ± 41.9	0.003
Fibroblast growth factor 21T120 (ng/mL)	194.84 ± 106.79	83.41 ± 46.05	<0.001
Ghrelin T0 (pg/mL)	475.51 ± 197.06	753.18 ± 261.2	<0.001
Ghrelin T60 (pg/mL)	444.87 ± 118.33	629.75 ± 207.15	<0.001
Ghrelin T120 (pg/mL)	450.66 ± 135.7	632.45 ± 203.07	<0.001
Glucagon-like peptide-1 T0 (nmol/dL)	0.74 ± 0.29	1.35 ± 0.36	<0.001
Glucagon-like peptide-1 T60 (nmol/dL)	0.67 ± 0.25	1.26 ± 0.33	<0.001
Glucagon-like peptide-1 T120 (nmol/dL)	0.68 ± 0.26	1.3 ± 0.38	<0.001
Leptin T0 (ng/dL)	34.84 ± 24.01	4.75 ± 4.52	<0.001
Leptin T60 (ng/dL)	34.93 ± 24.97	4.35 ± 4.7	<0.001
Leptin T120 (ng/dL)	30.94 ± 21.11	3.73 ± 3.68	<0.001
Leptin receptor T0 (µg/L)	8.21 ± 3.12	15.59 ± 6.49	<0.001
Leptin receptor T60 (µg/L)	8.4 ± 3.28	15.62 ± 6.78	<0.001
Leptin receptor T120 (µg/L)	8.47 ± 3.16	15.41 ± 6.23	<0.001
Resistin T0 (ng/mL)	3.65 ± 2	4.1 ± 1.18	0.0596
Resistin T60 (ng/mL)	3.5 ± 1.69	4.16 ± 1.05	0.023
Resistin T120 (ng/mL)	3.23 ± 1.25	3.85 ± 0.9	0.012
Visfatin T0 (ng/mL)	8.47 ± 6.9	17.49 ± 5.77	<0.001
Visfatin T60 (ng/mL)	8.66 ± 6.6	14.9 ± 3.44	<0.001
Visfatin T120 (ng/mL)	8.64 ± 5.77	12.95 ± 4.18	0.006

T0—measured at fasting; T60—measured at 60 min and T-120—measured at 120 min of the standard oral glucose tolerance test (OGTT).

**Table 3:** IGF-1, -2 and IGFBP-1, -2, -3, -4, -6, and -7 mean concentrations: comparison of obesity group (OB) and healthy control (HC). Values are presented as mean ± standard deviation.

Parameters	Obese Children (ng/mL)	Healthy Control (ng/mL)	*p* Value
IGF1	299 ± 112.9	231 ± 122.6	0.216
IGF2	51.95 ± 47.43	107 ± 43.86	0.06
IGFBP1	4.386 ± 5.66	7.85 ± 9.32	0.049
IGFBP2	75.62 ± 38.69	143.82 ± 124.387	0.021
IGFBP3	1896.46 ± 126.19	1763.88 ± 201.74	0.06
IGFBP4	54.1 ± 75.29	27.09 ± 19.17	0.02
IGFBP6	78.09 ± 28.45	151.9 ± 65.13	0.008
IGFBP7	42.25 ± 33.9	67.84 ± 48.47	0.06

**Table 4:** Correlation results of IGF1 and IGF2 concentrations with selected metabolic parameters.

	IGF1		IGF2	
Parameters	Spearman’s Correlation r	*p* Value	Spearman’s Correlation r	*p* Value
BMI percentile	0.239	0.22	−0.25	0.15
Blood pressure	0.089	0.689	−0.132	0.46
Insulin T0	0.179	0.37	−0.16	0.375
Insulin T60	0.249	0.219	−0.153	0.4
Insulin T120	0.283	0.152	−0.01	0.584
Adiponectin T0	−0.228	0.273	0.169	0.365
Adiponectin T60	−0.455	0.033	0.053	0.08
Adiponectin T120	−0.344	0.099	0.073	0.7
Apelin T0	0.124	0.547	0.388	0.028
Apelin T60	0.117	0.568	0.433	0.013
Apelin T120	0.213	0.3	0.374	0.035
Cholecystokinin T0	0.025	0.9	0.363	0.038
Cholecystokinin T60	0.16	0.423	0.49	0.004
Cholecystokinin T120	0.078	0.7	0.517	0.002
Fibroblast growth factor 21 T0	−0.3	0.1	−0.27	0.128
Fibroblast growth factor 21 T60	−0.29	0.14	−0.254	0.154
Fibroblast growth factor 21 T120	−0.257	0.2	−0.128	0.479
Ghrelin T0	−0.164	0.415	0.446	0.009
Ghrelin T60	0.042	0.836	0.44	0.01
Ghrelin T120	−0.327	0.513	0.459	0.007
Glucagon-like peptide-1 T0	0.117	0.569	0.366	0.04
Glucagon-like peptide-1 T60	0.043	0.83	0.429	0.013
Glucagon-like peptide-1 T120	0.058	0.773	0.5	0.002
Leptin T0	0.222	0.265	−0.4	0.02
Leptin T60	0.283	0.153	−0.45	0.008
Leptin T120	0.197	0.3	−0.45	0.007
Leptin receptor T0	−0.348	0.08	0.169	0.35
Leptin receptor T60	−0.338	0.08	0.127	0.483
Leptin receptor T120	−0.34	0.08	0.08	0.645
Resistin T0	0.21	0.3	0.227	0.212
Resistin T60	0.354	0.07	0.092	0.6
Resistin T120	0.085	0.672	0.234	0.189
Visfatin T0	−0.047	0.829	0.243	0.17
Visfatin T60	−0.066	0.749	0.28	0.12
Visfatin T120	−0.13	0.524	0.07	0.7

T0—measured at fasting; T60—measured at 60 min and T-120—measured at 120 min of the standard oral glucose tolerance test (OGTT).

**Table 5:** Correlation results of IGFBPs’ concentrations with selected metabolic parameters. T0—measured at fasting; T60—measured at 60 min and T-120—measured at 120 min of the standard oral glucose tolerance test (OGTT).

	IGFBP1		IGFBP2		IGFBP3		IGFBP4		IGFBP6		IGFBP7	
Parameters	Spearman’s Correlation r	*p* Value	Spearman’s Correlation r	*p* Value	Spearman’s Correlation r	*p* Value	Spearman’s Correlation r	*p* Value	Spearman’s Correlation r	*p* Value	Spearman’s Correlation r	*p* Value
BMI percentile	−0.34	0.051	−0.22	0.14	0.415	0.015	0.073	0.68	−0.417	0.014	−0.077	0.667
Blood pressure	−0.502	0.003	−0.39	0.006	0.39	0.02	0.217	0.219	−0.212	0.228	0.116	0.515
Insulin T0	−0.54	0.001	−0.343	0.02	0.4	0.02	0.022	0.905	−0.4	0.022	−0.186	0.299
Insulin T60	−0.493	0.004	−0.37	0.011	0.475	0.006	0.2	0.272	−0.33	0.06	−0.026	0.888
Insulin T120	−0.409	0.018	−0.3	0.02	0.367	0.036	0.256	0.15	−0.135	0.455	0.023	0.9
Adiponectin T0	0.194	0.295	0.076	0.62	−0.307	0.093	−0.186	0.315	0.206	0.266	−0.112	0.553
Adiponectin T60	0.157	0.424	0.076	0.617	−0.198	0.314	−0.224	0.25	0.163	0.407	−0.129	0.514
Adiponectin T120	0.144	0.449	0.04	0.743	−0.208	0.27	−0.188	0.319	0.173	0.36	−0.138	0.466
Apelin T0	0.193	0.3	0.122	0.286	−0.333	0.06	0.175	0.337	0.73	<0.001	0.356	0.046
Apelin T60	0.287	0.112	0.08	0.47	−0.363	0.04	0.085	0.64	0.636	0.00009	0.266	0.14
Apelin T120	0.23	0.207	0.087	0.45	−0.288	0.1	0.099	0.589	0.624	0.0001	0.253	0.16
Cholecystokinin T0	0.259	0.145	0.086	0.566	−0.459	0.007	−0.119	0.51	0.677	0.00001	0.304	0.3
Cholecystokinin T60	0.26	0.14	0.136	0.364	−0.278	0.117	−0.148	0.41	0.737	<0.001	0.163	0.365
Cholecystokinin T120	0.217	0.2	0.066	0.659	−0.358	0.04	0.01	0.956	0.653	0.00004	0.2	0.238
Fibroblast growth factor 21 T0	−0.2	0.266	−0.257	0.08	0.166	0.357	0.199	0.268	−0.474	0.005	0.16	0.37
Fibroblast growth factor 21 T60	−0.14	0.4	−0.27	0.066	0.234	0.195	0.203	0.258	−0.416	0.016	0.167	0.354
Fibroblast growth factor 21 T120	−0.05	0.777	−0.268	0.068	0.206	0.25	0.126	0.486	−0.349	0.046	0.11	0.54
Ghrelin T0	0.36	0.037	0.345	0.017	−0.14	0.426	−0.309	0.08	0.108	0.55	−0.126	0.486
Ghrelin T60	0.3	0.09	0.343	0.018	0.05	0.778	−0.355	0.04	0.223	0.2	−0.222	0.215
Ghrelin T120	0.29	0.1	0.364	0.012	−0.009	0.96	−0.294	0.01	0.22	0.219	−0.166	0.355
Glucagon-like peptide-1 T0	0.282	0.118	−0.032	0.84	−0.35	0.049	−0.02	0.909	0.745	<0.001	0.259	0.15
Glucagon-like peptide-1 T60	0.299	0.09	0.087	0.56	−0.3	0.026	0.05	0.785	0.677	0.00002	0.319	0.07
Glucagon-like peptide-1 T120	0.234	0.182	0.048	0.748	−0.372	0.033	0.137	0.448	0.7	<0.001	0.374	0.032
Leptin T0	−0.409	0.018	−0.326	0.025	0.17	0.343	0.029	0.873	−0.286	0.1	−0.025	0.89
Leptin T60	−0.407	0.019	−0.334	0.022	0.178	0.32	−0.0002	0.999	−0.287	0.1	−0.08	0.647
Leptin T120	−0.421	0.015	−0.328	0.025	0.156	0.386	−0.009	0.96	−0.3	0.078	−0.065	0.72
Leptin receptor T0	0.543	0.001	0.44	0.002	−0.634	0.001	−0.12	0.513	0.439	0.012	−0.165	0.367
Leptin receptor T60	0.524	0.002	0.427	0.003	−0.604	0.002	−0.147	0.4	0.444	0.01	0.14	0.427
Leptin receptor T120	0.518	0.002	0.455	0.002	−0.564	0.006	−0.157	0.38	0.454	0.008	0.12	0.502
Resistin T0	0.132	0.47	−0.055	0.716	−0.25	0.166	−0.078	0.67	0.148	0.419	0.087	0.635
Resistin T60	0.1	0.579	0.157	0.29	−0.34	0.052	−0.193	0.283	0.243	0.172	0.007	0.969
Resistin T120	0.42	0.015	0.108	0.469	−0.44	0.01	−0.29	0.1	0.237	0.184	−0.01	0.957
Visfatin T0	0.07	0.72	0.27	0.06	−0.26	0.14	0.384	0.03	0.568	0.0006	0.46	0.007
Visfatin T60	0.08	0.652	0.143	0.344	−0.2	0.24	0.493	0.004	0.353	0.047	0.502	0.003
Visfatin T120	0.248	0.17	0.094	0.536	−0.407	0.02	0.294	0.1	0.336	0.06	0.42	0.017

T0—measured at fasting; T60—measured at 60 min and T-120—measured at 120 min of the standard oral glucose tolerance test (OGTT).

**Table 6:** The expression values of IGF and IGFBP proteins’ genes.

Gene	Obese Children	Healthy Control	Fold Change	*p* Value
IGF1	5.15	5.63	−1.39	0.073
IGF2	6.09	6.49	−1.33	0.037
IGFBP1	7.28	7.91	−1.56	0.046
IGFBP2	6.4	6.65	−1.19	0.2
IGFBP3	6.12	6.25	−1.09	1.0
IGFBP4	7.68	7.81	−1.1	0.4
IGFBP5	5.01	5.1	−1.06	0.1
IGFBP6	6.83	7.22	−1.31	0.059
IGFBP7	8.08	7.37	1.64	0.023

## Data Availability

The datasets generated for this study are available on request to the corresponding author.
